# The effect of magnetic interference by magnetic resonance imaging on programmable shunt valves in patients with normal pressure hydrocephalus: a systematic review and pooled analysis

**DOI:** 10.1186/s12987-026-00837-y

**Published:** 2026-08-14

**Authors:** Virensinh Rathod, Darius Kalasauskas, Florian Ringel, Harroop Bola, Daniele S. C. Ramsay, Karanjot Chhatwal, Srikar R. Namireddy, Sree Kanakala, Iihan Ali, Dillon Nanoo, Eeshan Gangwani, Terrenjit Gill, Ayush Jha, Ahmed Salih, Madhur Varadpande, Abith G. Kamath, Ahkash Thavarajasingam, Dragan Jankovic, Andreas Kramer, Santhosh G. Thavarajasingam

**Affiliations:** 1 Imperial Brain & Spine Initiative, Imperial College London, London, UK; 2 Department of Neurosurgery, Universitätsmedizin Mainz, Mainz, Germany; 3 Faculty of Medicine, Imperial College London, London, UK; 4 Faculty of Medicine, University of East Anglia, London, UK; 5 Faculty of Medicine, King’s College London, London, UK; 6 Faculty of Medicine, Medizinische Hochschule Hannover, Hannover, Germany; 7 Department of Neurosurgery, LMU University Hospital, LMU Munich, Munich, Germany

## Abstract

**Introduction:**

Normal pressure hydrocephalus (NPH) is a cause of neurological impairment, with programmable shunt valves serving as the standard treatment. However, magnetic interference can result in altered pressure settings, affecting valve functionality.

**Research question:**

This systematic review investigates the effects of magnetic resonance imaging (MRI) on programmable shunt valves in NPH patients.

**Material and methods:**

Conducted in adherence to PRISMA guidelines, an extensive literature search across PubMed and Embase databases from January 2000 to January 2025, was performed. Studies evaluating the impact of MRI at 1.5T and 3T field strengths on valve settings were included. Valve reprogrammability and outcomes were analysed, with statistical comparisons across MRI strengths and valve types using Mann-Whitney U and ANOVA tests.

**Results:**

The pooled analysis included 475 NPH patients across 11 studies. Valve setting alteration was required in 44.2% of cases following 1.5T exposure and 55.2% after 3T exposure, with no statistically significant difference between field strengths (*p* > 0.05). Valve type analysis revealed that the Codman Hakim valve exhibited the highest incidence of setting changes (37.05% at 3T). However, differences in valve setting changes between manufacturers were not statistically significant (*p* > 0.05).

**Discussion and conclusion:**

Magnetic interference from MRI can disrupt programmable shunt valves in NPH patients. While 3T MRI shows a slightly higher incidence of valve setting changes than 1.5T, the difference is not significant. Further research is required to assess long- term clinical implications and standardize MRI protocols to mitigate variability in outcomes.

**Clinical trial number:**

Not applicable.

## Introduction

Idiopathic normal pressure hydrocephalus (iNPH) is characterised by Hakim’s clinical triad of gait disturbance, cognitive deterioration, and urinary incontinence, associated with ventriculomegaly in the context of normal cerebrospinal fluid (CSF) pressure [1–3]. The estimated pooled prevalence is 1.30% [4, 5], but this is likely an underestimate, due to frequent misdiagnosis with Parkinsonian disorders and concomitant dementia [6]. Magnetic resonance imaging (MRI) is frequently used in the diagnosis of NPH, evaluating radiological markers such as Evan’s index and the callosal angle, which have been demonstrated to be effective predictors in response to treatment [7–9].

Since their introduction in 1956, the surgical implantation of ventriculo-peritoneal and lumbo- peritoneal shunts remain the gold standard treatment [10–13]. These shunt systems effectively divert excess CSF from the cerebral ventricles to the intraperitoneal space, reducing intracranial pressure and restoring physiological CSF circulation. This is effective in improving Hakim’s clinical triad in approximately 60–80% of patients [14, 15]. The cumulative complication rate is approximately 32%, predominantly attributed to over drainage, manifesting as non-traumatic subdural haematomas [16–18]. The introduction of programmable shunt valves has improved the rates of these overdrainage-related complications, as compared with conventional fixed- differential pressure valves [19]. Programmable shunt valves can non-invasively modulate CSF drainage rates using magnetically activated components governing adjustable opening pressure settings. These settings dictate the precise threshold at which the valve permits CSF drainage, as per the specific individualised needs of each patient [20].

There is evidence to suggest that magnetic interference in MRI scans can affect valve programmability and positioning, potentially worsening clinical outcomes. Non-human laboratory studies found resistance to inadvertent valve pressure changes from MRI exposure at field strengths up to 3 Tesla (T), but greater field strengths resulted in perturbations to valve programmability and positioning [21]. An in-vitro study by Chen et al., revealed the loss of pressure setting re-programmability in CODMAN CERTAS^®^ Plus programmable VP-shunt valves coupled with susceptibility to ferromagnetic critical deflection when subjected to 7 T whole-body MRI systems [22]. These findings corroborate with an in-vivo single-centre prospective study by Capitanio et al., that reported a global alteration rate of 40% in pressure valve settings as a consequence of 1.5 T MRI on Hakim-Codman programmable valves [23]. Likewise, a series of case reports and in-vitro investigations have yielded comparable alterations in programmable valves when subjected to external magnetic field emitting devices including smartwatches, smartphones, and to a lesser effect, osseointegrated hearing devices [24–27].

Despite these reports, there remains no consensus on the real-world MRI safety of programmable valves. Evidence of valve-setting alterations is scattered across small in-vivo studies and case reports, and no synthesis currently exists to quantify the effect of MRI on shunt valve settings. The present systematic review aims to evaluate in-vivo evidence on the direct effect of magnetic interference on programmable shunt valves in patients with NPH.

## Methodology

### Search strategy and study selection

**Fig. 1:**
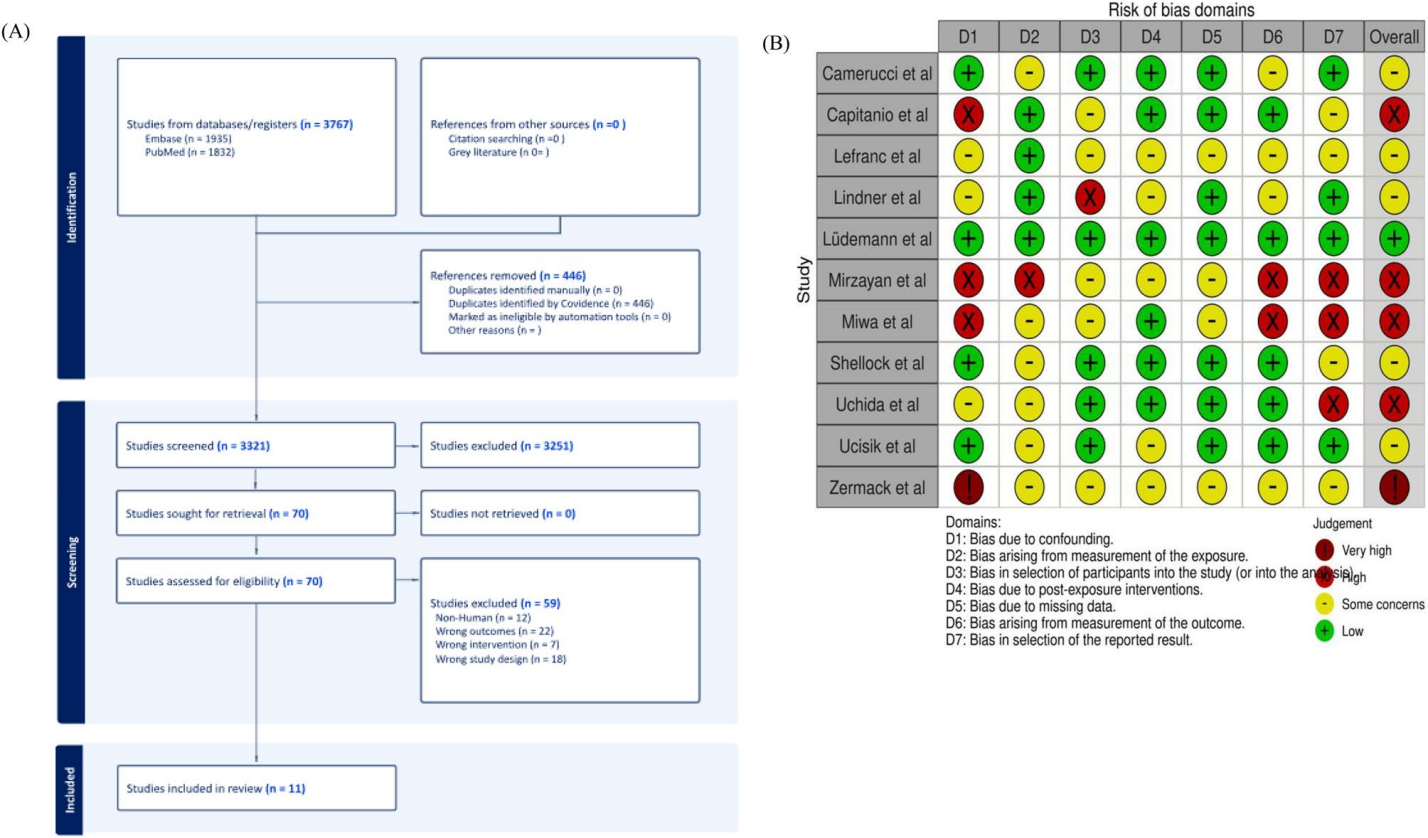
In **A**, the Preferred Reporting Items for Systematic Reviews and Meta-Analyses (PRISMA) flowchart outlining the study selection process is shown. Each step corresponds directly to the study selection process: records identified through database searching, removal of duplicates, screening of titles and abstracts, assessment of full-text eligibility, and final inclusion of studies meeting the predefined criteria. In **B**, a risk of bias summary plot for studies is presented, using a bar chart of the distribution of risk-of-bias judgments for all included studies across the domains of the Risk of Bias in Non-Randomised Studies of Interventions (ROBINS-E) tool: Bias due to confounding (Domain 1), Bias in selection of participants (Domain 2), Bias in classification of interventions (Domain 3), Bias due to deviations from intended interventions (Domain 4), Bias due to missing data (Domain 5), Bias in measurement of outcomes (Domain 6), Bias in selection of the reported result (Domain 7). An overall risk-of-bias score, representing the collated judgments for all domains, is displayed at the bottom for each plot. Percentages (%) are depicted for clarity

This study was registered on PROSPERO (CRD42024502448) on 20/01/2025 and was conducted in accordance with PRISMA 2020 guidelines [28]. The literature search, conducted on January 26th, 2025, across PubMed and Embase aimed to identify studies evaluating the impact of MRI at 1.5T and 3T field strengths on valve settings. The complete search strategy is detailed in Table 1, and the PRISMA flowchart summarizing the study selection process is presented in Fig. 1A. Only original, quantitative, peer-reviewed studies published in English that examined the longitudinal efficacy of MRI field strengths in adult populations were included. Eligibility criteria, based on the PICOS framework, focused on studies analysing magnetic field strengths for patients with NPH with outcomes such as percentage of valves affected and requiring reprogramming. Only primary studies were included. Exclusion criteria included reviews, abstracts, editorials, and case reports, with full details provided in Table 2 Initial title and abstract screening were conducted using Covidence software, enabling duplicate removal and independent review by five authors (DN, EG, TG, AJ, IA) [29]. Full-text articles were subsequently assessed by the same reviewers, with any disagreements resolved through consensus.

**Table 1:** Search strategy

Database	Search terms	Publication dates	Results (*n*)
Pubmed, Embase	(‘Magnet*’ OR ‘Magnetic field’ OR ‘Electromagnetic fields’ OR ‘Magnetics’ OR ‘Magnetism’) AND (‘programmable shunt valves’ OR ‘cerebrospinal fluid shunts’ OR ‘VP shunt’ OR ventriculoperitoneal shunt’ OR ‘shunt*’) AND (‘hydrocephalus’ OR ‘hydrocephalic’ OR ‘NPH’ OR ‘normal pressure hydrocephalus’ OR ‘Normal-pressure hydrocephalus’) AND (‘Phone*’ OR ‘Mobilephone’ OR ‘smartphone’ OR ‘iPhone’ OR ‘cellphone’ OR ‘mobile device’ OR ‘wireless device’ OR ‘audio device’ OR ‘handset’ OR ‘hearing device’ OR ‘hearing aid’ OR ‘cochlear implant’ OR ‘audio processing device’ OR ‘assistive listening device’ OR ‘personal sound amplifier’ OR ‘communication device’ OR ‘audio equipment’ OR ‘Magnetic resonance imaging’ OR ‘MRI’ OR ‘magnetic resonance*’ OR ‘magnetic stimulation.	2000–2025	*n* = 3,769

In Table 1 the search strategy performed on 26th of January 2025 is shown below outlining the respective databases, the search terms, publication dates chosen as limiting factors, and number of results from each database are shown

**Table 2:** Inclusion and exclusion criteria

Inclusion criteria	Exclusion criteria
- Hydrocephalus patients implanted with externally programmable cerebrospinal fluid (CSF) shunt systems - Any age - Any gender - Children and adults - Magnetic field strength - Magnetic resonance imaging - Magnetic fields - Exposure to magnetic fields from various sources, including external magnets or magnetic devices (MRIs, Smartphone, hearing devices etc.) - Unintended alterations in valve settings, shifts, or movements induced by exposure to magnetic fields. - Primary study	- Non-English language - Mixed cohort unable to have results separated - Systematic review or meta-analysis - Editorial, case report, opinion article, letter, conference abstract, correction - Full text unavailable - Does not assess valve repositioning, valve settings, or valve alterations.

In Table 2, the inclusion and exclusion criteria used when filtering studies based off search results are shown

### Data extraction

Relevant data from each included study was manually extracted using COVIDENCE, capturing information on study characteristics (title, authors, country of publication, publication date), percentage of valves requiring setting alteration, demographic details and study conclusions [29]. For studies with missing data, corresponding authors were contacted for clarification.

### Critical appraisal

Five independent reviewers (DN, EG, TG, AJ, IA) evaluated the risk of bias for each included study using the Risk of Bias in Non-randomized Studies - of Exposures (ROBINS-E) tool which assesses potential biases across multiple domains [30]. The ROBINS-E tool assesses bias across seven domains, including confounding, participant selection, intervention classification, deviations, missing data, outcome measurement, and selective reporting. Discrepancies in assessments were resolved through consensus by a sixth reviewer.

### Statistical analysis

Quantitative analyses were performed to evaluate the rates of valve setting alteration across varying MRI field strengths, specifically at 1.5 Tesla (1.5T) and 3 Tesla (3T). These rates were further stratified by shunt valve manufacturer to identify potential differences in performance or susceptibility to reprogramming effects. For inter-group comparisons, non-parametric tests such as the Mann-Whitney U test were employed for two-group comparisons, particularly when the data did not meet assumptions of normality. To assess differences across multiple groups, analysis of variance (ANOVA) was utilized, with post hoc testing conducted where appropriate to determine pairwise significance. We performed additional random-effects meta-analyses to obtain pooled estimates of 1.5T and 3T reprogramming rates, excluding zero-event studies. A paired random-effects risk ratio analysis was conducted using only studies that reported both 1.5T and 3T outcomes. All meta-analysis was performed using the *meta* package.

Descriptive statistics, including mean values and standard deviations for continuous variables, as well as percentages for categorical variables, were calculated to summarize the aggregated dataset effectively. Graphical visualizations were also generated to illustrate the distribution of reprogramming rates across different variables.

All statistical analyses were conducted using R software (version 4.4.3), ensuring reproducibility and adherence to robust analytical frameworks. A *p*-value threshold of < 0.05 was set to determine statistical significance, with confidence intervals (95%) reported for key comparative outcomes to provide a measure of precision around the estimates. For consistency across studies, a valve ‘setting alteration’ was defined as any measurable change in programmed opening pressure between pre-MRI and post-MRI assessment.

## Results

A total of 3,321 studies were screened, of which 3,251 were excluded based on titles and abstracts, leaving 70 full-text articles assessed against the inclusion and exclusion criteria. Ultimately, 11 studies met the criteria and were included in this systematic review (Fig. 1A). These studies encompassed a combined sample of 475 patients. Study characteristics, including study design, country of origin, sample size, follow-up duration, and intervention type, are detailed in Tables 3 and 4. The risk of bias was assessed using the ROBINS-E tool. In the ROBINS- E tool, 4 studies were rated as “serious” overall (Fig. 1B).

**Table 3:** Study characteristics of the included studies in this systematic review and pooled analysis

Authors	Study type	Patient population	M: F	Median age	Sample size	MRI Tesla	Shunt type	Endpoints Assessed	Study Protocol
Camerucci et al. [36]	Prospective cohort	iNPH patient	18:04	73.2	22	1.5 and 3.0	CERTAS Plus valve	Artifact Extent, Full Cohort; Patients with both 1.5 and 3T MRIs on either GE or Siemens; concordance amongst reviewers	0.5-year duration from MRI to MRI
Capitaniol [23]	Prospective cohort	Children + adult hydrocephalus	N/A	N/A	100	1.5	Codman Hakim programmable valve	Number and rate of changes in valve before and after MRIexposure; rate of deregulation	T1-weighted sequences, TR 596 mseconds, TE 15 mseconds, and FOV 23 mm (288 matrix); T2- weighted Turbo spin Echosequence, TR 4454 mseconds, TE 100 mseconds, FOV 230 mm (480 matrix); and diffusion-weighted single shot sequences with enhanced gradient mode and sense, TR 2929 mseconds, TE 62 mseconds, FOV 240 mm (288 matrix).
Uchida et al. [37]	Control trial	Not Hydrocephalus	N/A	N/A	1	3	4 adjustable shunt valves: proGAV 2.0 (Miethke), Codman Certas Plus (Johnson & Johnson), Polaris (Sophysa), and Strata MR valve (Medtronic)	Pressure settings of valves; size and shape of artifacts	Parameters given in table-outine brain MR images were obtained with the following sequences: T1- weighted imaging (T1WI); T2- weighted imaging (T2WI); fluid- attenuated inversion recovery (FLAIR); diffusion-weighted imaging (DWI); and 3D MR angiography (3D- MRA)
Ucisik et al. [38]	Retrospective cohort	Hydrocephalus patients	111:45:00	34(66 pediatrics, 144 adult)	156 patients but 210 exposure events	1.5 and 3.0	Codman Hakim™ (*n* = 102), Medtronic Strata NSC™ (*n* = 54), and Medtronic Strata II™ (*n* = 46).	Number of valve settings change; setting change rate based on MRI used	
							Other valves were much less common (*n* < 10) and included Aesculap ProGAV™, Polaris		
							Sophysa™, Codman Certas™, and Medtronic StrataMR™ valves.		
Zemack et al. [39]	.	NPH- 190 programmable valves in 174 patients	.	.	583 patients (421 adults and 162 children)	3	Codman Hakim programmable valve	Number of patients in whom valve opening pressure adjustment was required at least once; change in opening pressure at baseline and follow-up;	
Lindner et al. [40]	Control trial	Ex-vivo	N/A	N/A	9	3	Paedi GAV, dual switch and proGAV	Hydrodynamic Performance: Before and after exposure to MRI. Functional Stability: Adjustments in valve opening pressure. Effects of Static Magnetic Field: Influence on valve settings and functionality.	
Shellock et al. [41]	Control trial	in vitro	N/A	N/A	9	3	Codman Hakim Programmable Valve:	Magnetic Field Interactions: Deflection	1 min each, 8 Pulse sequences
							Magnetically programmable valve for cerebrospinal fluid (CSF) management. Adjustable pressure range: 30 to 200 mm H₂O in 10 mm H₂O increments. Materials: Stainless steel, unalloyed titanium, and magnetic alloys (Vacoperm, Vacomax).		
Mirzayan et al. [42]		Ex-vivo			20 valves for 3T, 20 valves for 7T. 0 patients	3 and 7	Programmable shunt (proSA)	The deflection angle toward the MRI was measured three times and then averaged.	
Miwa et al. [43]	Prospective cohort	Hydrocephalus patients	05:02	65	7 cases but 72 tests for R retroauricular placement and 72 tests for anterior chest wall placement	0.3	Codman- Medos	Changes in valve setting after MR exposure; changes to pressure setting after flipping of valve	
Lüdemann et al. [44]	Comparative study	Ex-vivo	N/A	N/A	10 adjustable shunt devices but 340 examinations in total (34 MRI examinations for each device)	3	Polaris SPV	Changes in the adjusted pressures	
Lefranc et al. [45]	Control trial	In-vitro	N/A	N/A	4 valves and 3 experiments	Transcranial magnetic stimulation performef with a Magpro X100 stimulator, with a twin mode of up to 1.5T for double shocks and a power mode of up to 2.0T for other patterns of magnetic stimulation	Strata 2 from Medtronic, Polaris from Sophysa, ProGAV from Miethke and a cylindrical valve from Codman- Hakim	Experiment 1: temperature and opening pressure of each valve before and after TMS after each test, the direction and extent of the pressure changes were determined visually experiment 2: valve temperature changes before and after magnetic stimulation opening pressure before and after TMS after each test, the direction and extent of pressure changes determined visually	
								Experiment 3: movement of valves by measurement of acceleration amplitudes using signal from accelerometer	

In Table 3, the study characteristics of all included studies are presented, detailing the study type, patient population, male: female ratio, age, sample size, MRI tesla, shunt type, endpoints assessed and study protocol

**Table 4:** Reprogramming rates for individual programmable valve types across included studies

Study	Codman		Miethke		Medtronic			Sophysa
	Hakim	CERTAS plus	proGAV 2	proSA	Strata MR	Strata II	Strata NSC	Polaris
Zemack et al. [39]	42.5%	–	–	–	–	–	–	–
Ucisik et al. [38]	32.4%		85.2%	–		–	–	87.0%
Uchida et al. [37]	–	0%	0%	–	0%		–	0%
Capitanio et al. [23]	40.0%	–	–	–	–	–	–	–
Camerucci et al. [36]		27.2%	–	–	–	–	–	–
Lindner et al. [40]	–	–	0%	–	–	-	0%	–
Shellock et al. [41]	67.0%	–	–	–	–	–	–	–
Mirzayan et al. [42]	–	–	–	0%	–	–	-	–
Miwa et al. [43]	80.6%	–	–	–	–	–	–	–
Lüdemann et al. [44]	–	–	–	–	–	–	-	0%
Lefranc et al. [45]	28.0%	–	0%	–	–	24.0%	-	0%

Values represent the proportion of valves requiring post-MRI reprogramming, highlighting substantial variability in susceptibility between valve models

### Valve pressure setting changes

The primary outcome across studies was the assessment of valve settings following MRI exposure. We looked for changes in valve pressure settings. Variance in the marker is seen via Capitanio et al. examining the number and rate of valve setting changes before and after MRI exposure, alongside deregulation rates.

Zemack and Romner analysed the proportion of patients requiring at least one valve opening pressure adjustment post-MRI, finding that patients who underwent shunt revision had a higher reprogramming rate (48%) than those with initially implanted valves (41.2%). Among NPH patients, 42.5% required reprogramming at least once, with a mean pressure change of 15 mmH₂O at follow-up compared to the initial shunt placement. Accidental resets exceeding 20 mmH₂O occurred in 20.1% (35/174) of cases. Figure 3 illustrates the distribution of valve setting changes across different brands. The study noted more generally that MRI caused a resetting of the pressure valve in 11/41 (26.8%) cases in patients whose valves were implanted in 1997; not exclusively to NPH. Miwa et al. reported that 80.6% of valves experienced pressure setting alterations after 0.3T MRI exposure in the retroauricular placement.

### Valve reprogramming following MRI exposure

**Fig. 2:**
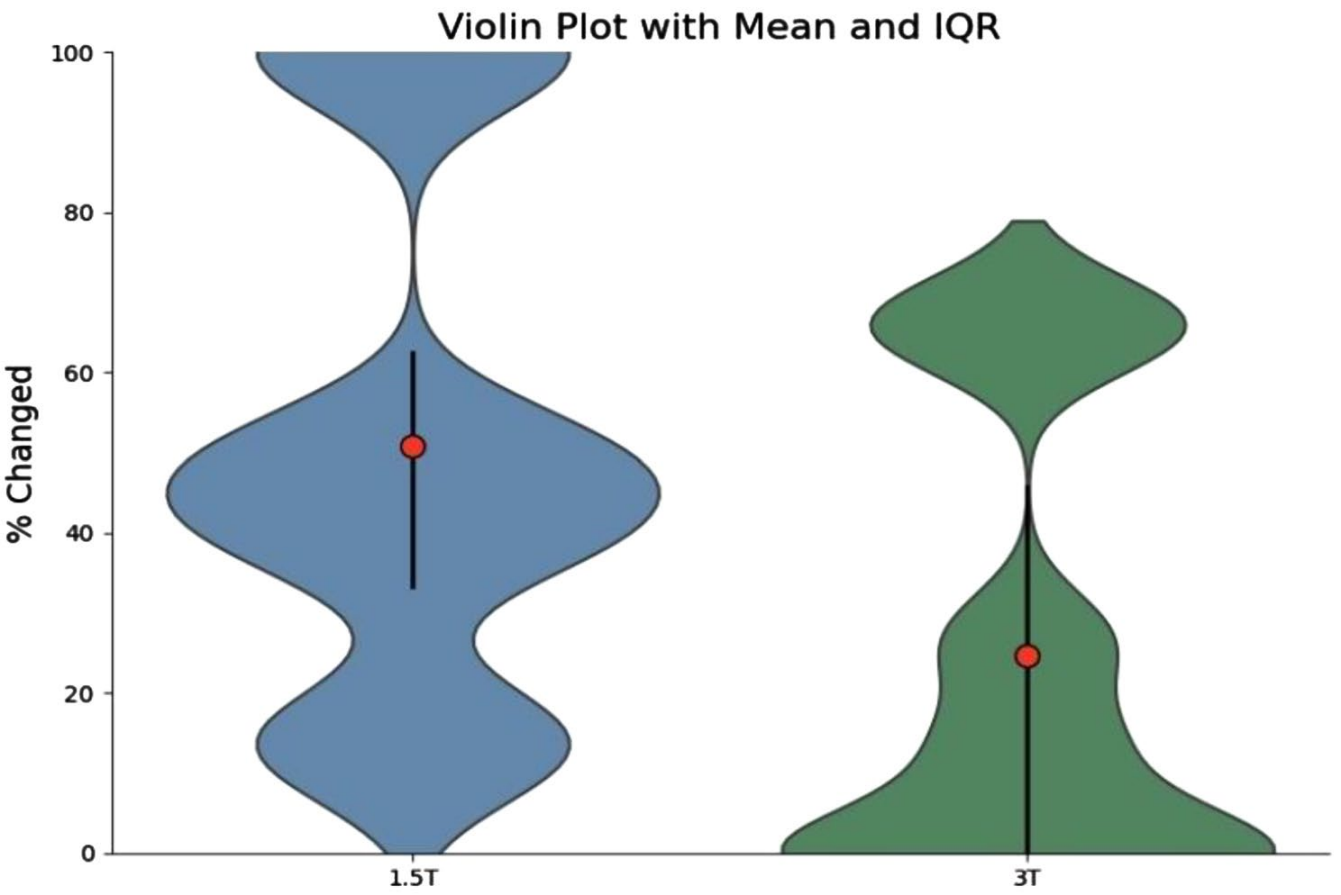
Violin plot illustrating the percentage change in valves for Group 1.5T (represented in blue) and Group 3T (represented in green). Kernel density estimation (KDE) is applied, with the distribution range constrained between 0% and 100% to ensure that only positive percentage changes are depicted, reflecting absolute values of valve setting change. The width of each violin represents the frequency of observed values, with wider regions indicating higher data density. The red dot represents the mean percentage change, and the vertical black line denotes the interquartile range (IQR), providing a measure of variability around the median

Figure 2 illustrates a grouped analysis, demonstrating that 44.2% of valves were affected at 1.5T, while 55.2% were affected at 3T. However, statistical comparison using the Mann-Whitney U test (*p* = 0.2514) revealed no significant difference between these field strengths.

Studies conducted by Uciisk et al. and Camerucci et al. directly compared the impact of 3T versus 1.5T under identical conditions, using the Wilcoxon signed-rank test, which similarly demonstrated no significant difference (*p* > 0.05). The percentage of affected valves was 44.2% at 1.5T and 55.17% at 3T, reflecting an absolute difference of 10.97%, though this was not statistically significant.

**Fig. 3:**
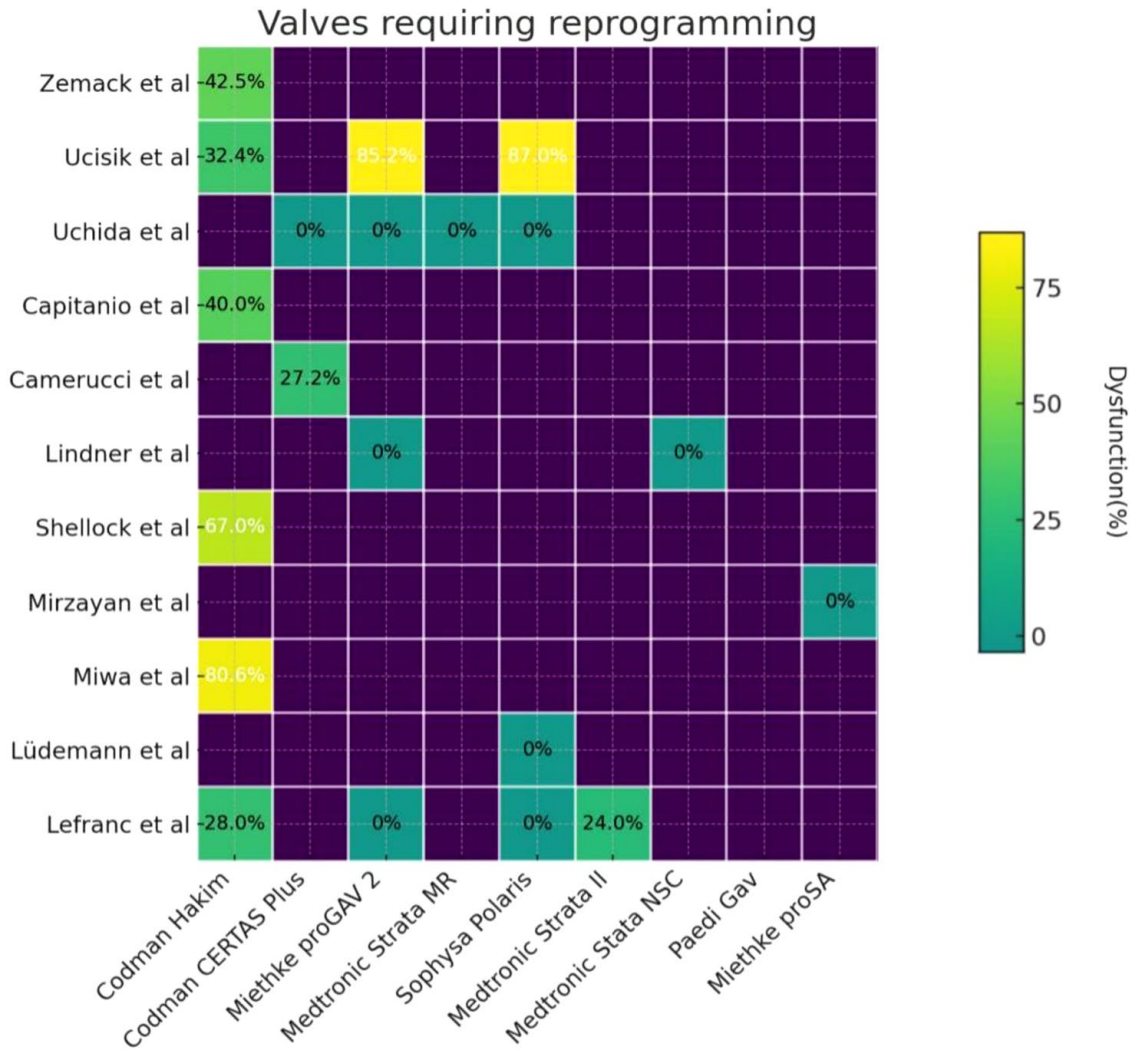
Heatmap depicting valve reprogramming status by author, showing the percentage of valves affected by dysfunction post- reprogramming. Data reported is raw data% within each study

Figure 3 provides a breakdown of valve setting alteration rates by brand following MRI exposure. The analysis of valve setting alteration rates across multiple studies revealed significant variability among different programmable shunt valves. The highest individual rates of dysfunction were reported by Ucisik et al., with Mietke proGAV 2 (85.2%) and Medtronic Strata MR (87.0%) demonstrating the greatest susceptibility to external interference. Similarly, Miwa et al. reported an 80.6% dysfunction rate, while Shellock et al. and Zemack et al. observed rates of 67.0% and 42.5%, respectively. In contrast, several studies, including those by Uchida et al. and Lindner et al., reported 0% dysfunction for multiple valve models, suggesting greater resistance to external influences. Medtronic Strata NSC (24.0%) demonstrated a moderate reprogramming rate in the study by Lefranc et al., whereas Codman CERTAS Plus, Sophysa Polaris, and Medtronic Strata II exhibited no dysfunction across multiple reports. These findings highlight substantial differences in valve susceptibility to setting alteration, emphasizing the need for careful selection based on clinical context and patient-specific factors.

### Valve specific variability

Analysis of valve manufacturers indicated variability in valve setting alteration rates. In pooled analysis of valve type, the Codman Hakim valve exhibited the highest setting alteration rate at 37.05%, followed by the Sophysa Polaris valve at 18.46% and the Miethke proGAV 2 valve at 17.98%. Statistical analysis using ANOVA revealed no significant difference in valve setting alteration rates across valve types (*p* > 0.05).

These findings suggest that while 3T MRI exposure resulted in a numerically higher rate of valve setting changes, direct paired comparisons and statistical analyses did not support a significant difference between 1.5T and 3T MRI in programmable valve dysfunction.

### Key findings & meta-analysis results

**Fig. 4:**
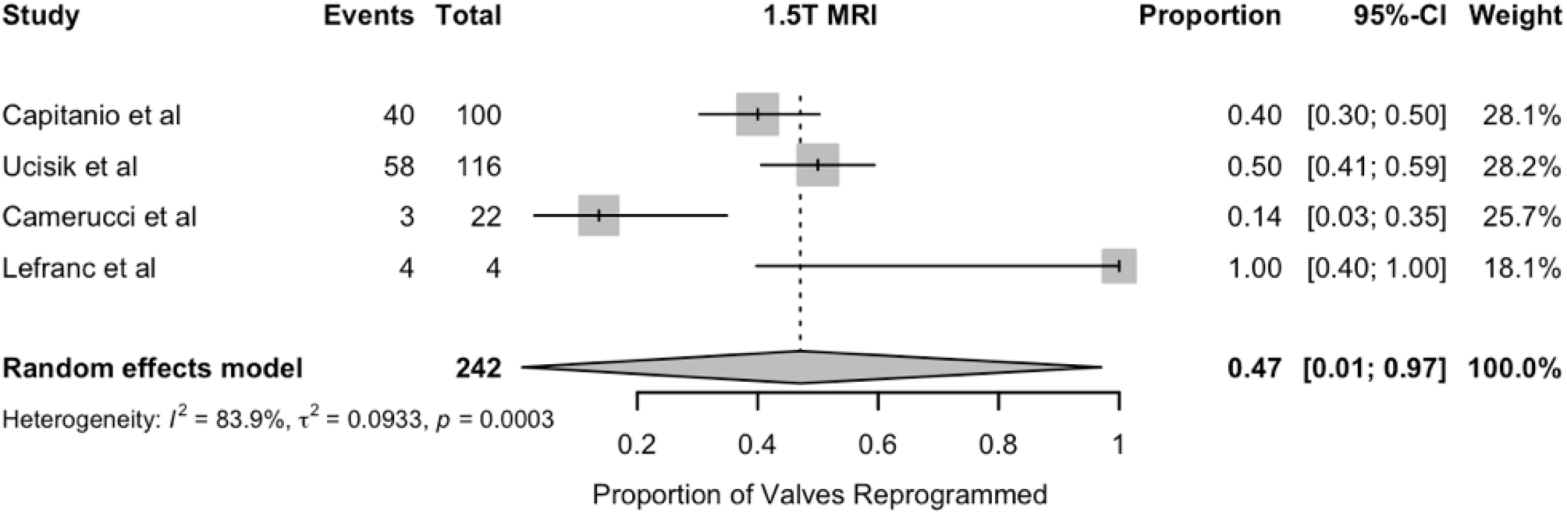
Random-effects meta-analysis of the proportion of programmable shunt valves exhibiting pressure setting changes following 1.5-Tesla MRI. Individual study estimates are shown with 95% confidence intervals, weighted by inverse variance using the Freeman-Tukey transformation. Zero-event studies were excluded. The pooled estimate was 0.47, with substantial heterogeneity (I² = 83.9%, *p* < 0.001)

**Fig. 5:**
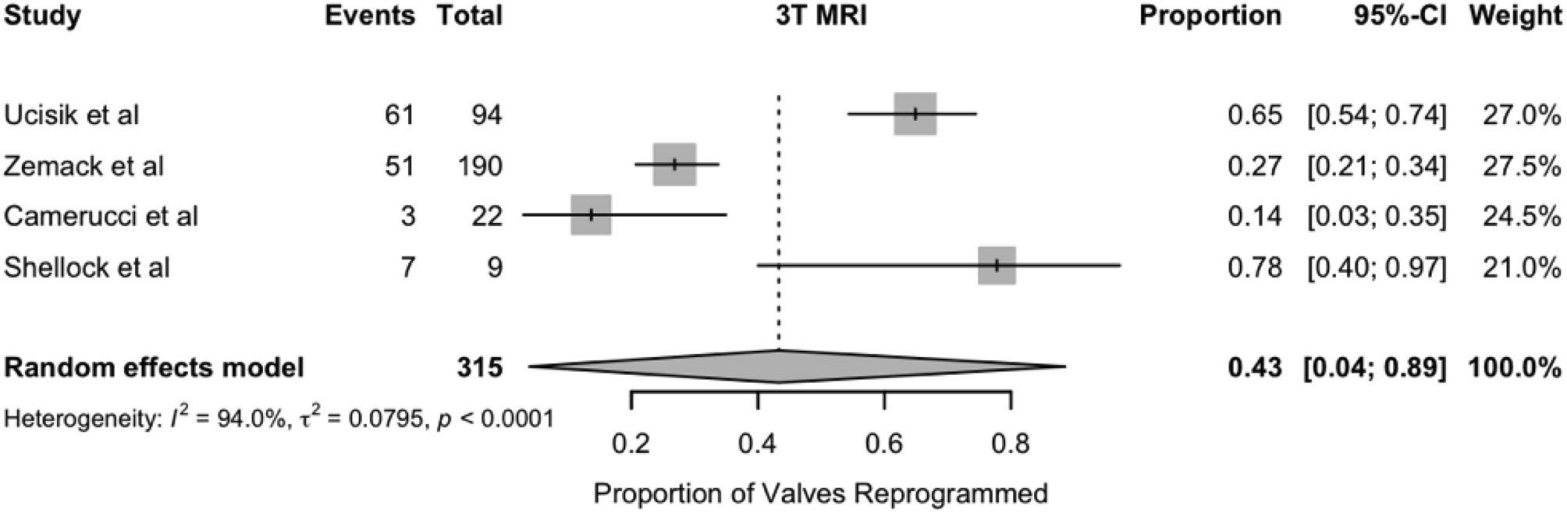
Random-effects meta-analysis of the proportion of programmable shunt valves exhibiting pressure setting changes following 3-Tesla MRI. Individual study estimates are shown with 95% confidence intervals, weighted by inverse variance using the Freeman-Tukey transformation. Zero-event studies were excluded. The pooled estimate was 0.43, with substantial heterogeneity (I² = 94.0%, *p* < 0.001)

**Fig. 6:**
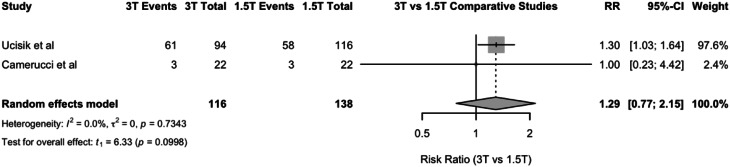
Random-effects risk-ratio meta-analysis comparing valve reprogramming rates between 3-Tesla and 1.5-Tesla MRI in the only two studies reporting outcomes at both field strengths within the same cohort. Individual study risk ratios with 95% confidence intervals are shown. The pooled risk ratio was 1.29 (95% CI 0.77, 2.15), indicating no statistically significant difference in reprogramming risk between 3T and 1.5T MRI

A total of 24.4% of programmable valves required reprogramming following MRI exposure. Among studies comparing different MRI field strengths, 44.2% of valves were affected at 1.5T, while 55.2% were affected at 3T, though this difference was not statistically significant. Codman Hakim valves exhibited the highest reprogramming rates, yet variability in valve response was noted across different brands. Random-effects meta-analysis demonstrated a pooled reprogramming rate of 47% (95% CI: 1%, 97%) at 1.5T and 43% (95% CI: 4%, 89%) at 3T, with high heterogeneity across studies (Figs. 4 and 5). To directly compare field strengths, only the two head-to-head studies were analysed using a paired random-effects risk ratio model, yielding an RR of 1.29 (95% CI 0.77, 2.15), indicating no statistically significant difference between 3T and 1.5T MRI (Fig. 6). These results suggest that variability between studies, rather than magnetic field strength alone, may explain differences in reported reprogramming rates.

### Risk of bias

Risk of bias was evaluated across seven ROBINS-E domains for all included studies (Fig. 1B). Four studies (Capitanio et al., Uchida et al., Mirzayan et al., and Miwa et al.) were judged to have a high or very high overall risk of bias, most commonly due to concerns relating to confounding, exposure classification, and outcome measurement. The remaining studies were rated as having low or some concern overall risk. To assess the influence of high-risk studies on the robustness of our findings, a sensitivity analysis excluding all high-risk-of-bias studies was performed. This exclusion did not meaningfully alter pooled reprogramming rates or the conclusions of the head-to-head analysis, indicating that the primary findings of this review are stable and not driven by studies at highest risk of bias.

## Discussion

This study provides a systematic evaluation of the impact of MRI exposure on programmable shunt valves, demonstrating that both 1.5T and 3T MRI induce valve setting changes. However, the difference in the proportion of affected valves between the two field strengths was not statistically significant, suggesting that while 3T MRI exposure results in numerically higher reprogramming rates, it does not necessarily pose a greater risk compared to 1.5T. The variability in reported outcomes may be attributed to study design heterogeneity, sample size discrepancies, differences in valve models and patient populations, and variations in MRI exposure conditions. Differences in imaging protocols and hardware configurations across studies may further contribute to inconsistencies in reprogramming rates, highlighting the necessity for standardised experimental methodologies.

The median percentage change in valve settings was 45.0% for 1.5T and 6.8% for 3T. This disparity may reflect the imbalance in the number of studies reporting on each field strength, with a greater representation of 3T MRI studies introducing a potential reporting bias. The findings suggest that while the aggregated analysis indicates a higher percentage of affected valves at 3T, paired comparisons under controlled conditions did not reveal a statistically significant difference. Findings of Lefranc et al. included results of 1.5T TMS in valve programmability but not within sub analysis of field strength.

Additionally, variations in study protocols, such as MRI sequence parameters, exposure durations, and spatial gradients of magnetic field intensity, likely contributed to inconsistencies in reported reprogramming rates. Given the limited availability of data for 1.5T MRI, further research is required to establish a more balanced dataset and enhance the reliability of comparative analyses. This disparity may be attributed to variability in the data, which can be explained by differences in study design, sample size and reporting cases. Notably, more studies reported data for 3T than for 1.5T, potentially introducing a reporting bias. The higher number of studies for 3T could reflect a growing preference for higher-field MRI systems in clinical practice, resulting in more available data for this subgroup. Having a more extensive evaluation of 1.5T MRI exposure across multiple programmable valve designs may help determine whether specific device characteristics or patient factors influence susceptibility to magnetic interference [31].

The placement of programmable valves within the cranial cavity is an important factor influencing in vivo reprogramming susceptibility [32]. The interaction of these devices with cerebrospinal fluid dynamics and adjacent brain structures may not be accurately replicated in ex vivo studies, underscoring the necessity for in vivo investigations [33]. Moreover, valve- specific differences in magnetic shielding mechanisms impact susceptibility to MRI-induced changes [31]. Certain models, such as the Miethke proGAV, incorporate locking mechanisms that confer resistance to unintentional reprogramming, explaining the observed stability in valve settings following 3T MRI exposure [34]. By contrast, Medtronic Strata II valves exhibited a higher frequency of setting alterations, aligning with prior studies highlighting their increased sensitivity to magnetic interference [35]. The effectiveness of these magnetic resistance mechanisms varies across manufacturers, further emphasising the importance of valve-specific assessments in determining MRI compatibility.

The findings also highlight the broader clinical implications of MRI-induced valve setting changes. Although alterations are typically reversible and reprogramming can be performed post-MRI, unintended changes in valve pressure settings may contribute to suboptimal cerebrospinal fluid drainage, potentially leading to clinical deterioration in vulnerable patients. Capitanio et al. reported a 40% deregulation rate following 1.5T MRI exposure, while Ucisik et al. found setting changes in 64.9% of cases at 3T, advocating for post-MRI valve verification to mitigate risks. Similarly, Zemack et al. emphasised the necessity for post-imaging radiographic assessment due to spontaneous valve resets in 26.8% of patients. Collectively, these findings reinforce the need for routine post-MRI valve setting evaluations to prevent adverse outcomes and ensure appropriate cerebrospinal fluid dynamics are maintained. Importantly, our findings also suggest that certain groups may warrant higher-priority post-MRI valve verification, including patients with Codman Hakim valves, which demonstrated the highest reprogramming rates, and those with recently implanted valves who may be more vulnerable to unintended setting shifts. Although data remain limited, these patterns provide preliminary suggestions that could inform future risk-stratified protocols for post-MRI valve assessment. Notably, older-generation valves appear more susceptible than newer MRI-resistant designs, which generally demonstrated low alteration rates across studies. Given the reliance on MRI for hydrocephalus monitoring, the integration of preemptive clinical guidelines for valve reprogramming following imaging procedures could mitigate risks associated with unexpected setting alterations. However, because most studies did not report clinical outcomes, the impact of unintended valve setting alterations, such as worsening symptoms, under- or over-drainage, or subdural collections, remains unknown. Future research should explore correlate valve setting changes with clinical outcomes, to determine the significance of MRI-induced reprogramming.

It should be further noted that valve manufacturers have released guidance regarding functionality under exposure to 3T or 1.5T, hence this study does not challenge stated specifications but rather aggregates the effect of MRI upon valve programmability. The implication is further seen by specification of Miethke^®^ stating non-clinical testing for MRI safety conditions. Furthermore, the lack of unified safety mechanisms regarding valve systems causes a bias to a greater degree in manufacturer rather than MRI strength. Hence explain a greater variance in study findings of individual study against valve type than aggregated analysis of MRI field strength.

This study has several limitations, including variability in study protocols, MRI exposure parameters, and timepoints for valve assessment. Many studies did not account for prior MRI exposure history or background magnetic field influences, introducing potential confounders. In addition, heterogeneity in how studies defined a ‘valve setting alteration’, ranging from any measurable pressure change to only threshold-based shifts, likely contributed to inter-study variability and should be considered when interpreting pooled reprogramming rates. Furthermore, the small number of studies exclusively reporting 1.5T MRI exposure limits direct comparison between field strengths. The lack of data on higher field strengths, such as 7T MRI, also prevents broader conclusions on the impact of ultra-high-field imaging on programmable valves. Additionally, differences in shunt valve positioning, patient head orientation within the scanner, and external electromagnetic field exposures may further influence reprogramming susceptibility, necessitating more controlled investigations. Regarding clinical relevance, the limitations of study and high bias suggest findings that do not challenge clinical safety. Furthermore, the lack of heterogeneity in endpoint assessment suggest that potential analysis in the percentage change of endpoints, would likely not yield findings to challenge legally stated manufacturer specifications in regards to tolerance.

## Conclusion

In conclusion, programmable shunt valves remain susceptible to MRI-induced setting changes, though difference between 1.5T and 3T MRI exposure is not statistically significant. While certain valve models demonstrate greater resistance to magnetic interference, routine post-MRI valve setting verification remains essential to prevent unintended pressure alterations. The variability in reprogramming rates across studies suggests that differences in imaging protocols, valve designs, and patient factors may all contribute to magnetic susceptibility. Further research, particularly involving in vivo studies, is necessary to refine MRI safety protocols, improve clinical outcomes for patients with hydrocephalus managed with programmable shunts, and establish evidence-based guidelines for pre- and post- imaging valve management.

## Data Availability

All relevant data supporting the findings of this study can be accessed within the Supplementary Digital Content attached to the article.
